# Characterization of the Specific CD4^+^ T Cell Response against the F Protein during Chronic Hepatitis C Virus Infection

**DOI:** 10.1371/journal.pone.0014237

**Published:** 2010-12-06

**Authors:** De-Yong Gao, Gen-Di Jin, Bi-Lian Yao, Dong-Hua Zhang, Lei-Lei Gu, Zhi-Meng Lu, Qiming Gong, Yu-Chun Lone, Qiang Deng, Xin-Xin Zhang

**Affiliations:** 1 Department of Infectious Diseases, Ruijin Hospital, Shanghai Jiaotong University School of Medicine, Shanghai, China; 2 Songjiang Hospital Affiliated to Shanghai First People's Hospital, Shanghai Jiaotong University, Shanghai, China; 3 INSERM U 542, Hôpital Paul Brousse, Villejuif, France; 4 Unit of Tumor Virology, Institut Pasteur of Shanghai, Chinese Academy of Sciences, Shanghai, China; University of Toronto, Canada

## Abstract

**Background:**

The hepatitis C virus (HCV) Alternate Reading Frame Protein (ARFP or F protein) presents a double-frame shift product of the HCV core gene. We and others have previously reported that the specific antibodies against the F protein could be raised in the sera of HCV chronically infected patients. However, the specific CD4^+^ T cell responses against the F protein during HCV infection and the pathological implications remained unclear. In the current study, we screened the MHC class II-presenting epitopes of the F protein through HLA-transgenic mouse models and eventually validated the specific CD4^+^ T cell responses in HCV chronically infected patients.

**Methodology:**

DNA vaccination in HLA-DR1 and-DP4 transgenic mouse models, proliferation assay to test the F protein specific T cell response, genotyping of Chronic HCV patients and testing the F-peptide stimulated T cell response in the peripheral blood mononuclear cell (PBMC) by *in vitro* expansion and interferon (IFN)- γ intracellular staining.

**Principal Findings:**

At least three peptides within HCV F protein were identified as HLA-DR or HLA-DP4 presenting epitopes by the proliferation assays in mouse models. Further study with human PBMCs evidenced the specific CD4^+^ T cell responses against HCV F protein as well in patients chronically infected with HCV.

**Conclusion:**

The current study provided the evidence for the first time that HCV F protein could elicit specific CD4^+^ T cell response, which may provide an insight into the immunopathogenesis during HCV chronic infection.

## Introduction

Over 170 million people worldwide are chronically infected with HCV. The chronic hepatitis C often results in cirrhosis of the liver and increases the probability of developing hepatocellular carcinoma [1], [2]. There is no HCV vaccine available so far [3] despite the fact that the combination of PEG-IFN-a and ribavirin is at present a standard regimen used for treating hepatitis C patients [4].

Cellular immune responses, involving both CD8^+^ cytotoxic T lymphocytes (CTLs) and CD4^+^ T-helper lymphocytes (HTLs), play an essential role in the control of HCV infection, as they do in other persistent viral diseases. Whereas CTLs are traditionally thought to be the main effector cells that eliminate HCV-infected cells [5], it is clear that HCV-specific CD4^+^ T cells also play a critical role. A growing body of evidence indicates that spontaneous clearance of HCV is associated with a strong HCV-specific proliferative CD4^+^ Th cell response. A number of studies on persistent murine and human viral infections indicate that virus specific CD4^+^ T cells play a critical role in the outcome of viral infections [6], [7], [8], [9], [10], and are required to maintain effective cytotoxic T cell responses [11] and neutralizing antibodies [12]. Notably, incomplete control of HCV replication due to inadequate CD4^+^ T cell help is usually associated with the emergence of viral escape mutation epitopes.

HCV alternate reading frame protein (ARFP/F) of the 1b genotype is a double-frame shift product of the HCV core gene [13], [14], [15]. It has been demonstrated that HCV F protein could elicit a specific antibody response other than the anti-core protein response [16], . The presence and the level of anti-F antibody response could be induced by interferon plus ribavirin treatment and associated with sustained virological response (SVR) in hepatitis C patients [17]. The current study was designed to comprehensively determine the specific CD4^+^ T cell responses in a cohort of patients with diverse HLA backgrounds, in order to understand the potential helper T cell response against HCV F protein during chronic HCV infection.

## Results

### Expression and Identification of the HCV F proteins in cultured cell line

**Figure 1:**
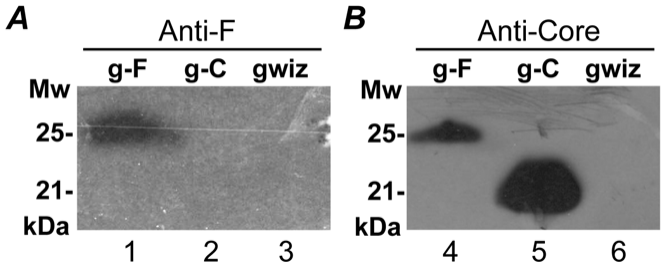
Expression of the HCV F protein after transient transfection. Three days after the transfection, Huh 7 cells were lysated for western blot analysis of the recombinant HCV F and core proteins with monoclonal anti-F (**A**) or anti-core (**B**) antibodies respectively. gWiz empty plasmid was used as the negative control for Huh 7 cell transfection. g-F, gWiz-F; g-C, gWiz-Core.

HCV F protein is composed of a central frameshift F domain (amino acids [aa] 43–144, genotype 1b) flanked by N-terminal and C-terminal fragments from HCV core protein. Expression of the F protein was studied with gWiz-F, a plasmid bearing the chimeric F gene under the control of cytomegalovirus early gene promoter. After transient transfection of gWiz-F to human hepatoma cell line Huh 7, the expression of HCV F protein was identified in cell lysates with its expected size (25 KDa) by western blot using specific anti-HCV core and anti- HCV F antibodies (**Fig. 1**). HCV F protein can also be recognized by anti-HCV core antibody, but with less intensity (**Fig. 1B**).

### HCV F protein activates specific CD4^+^ T cell response in HLA transgenic mice

**Figure 2:**
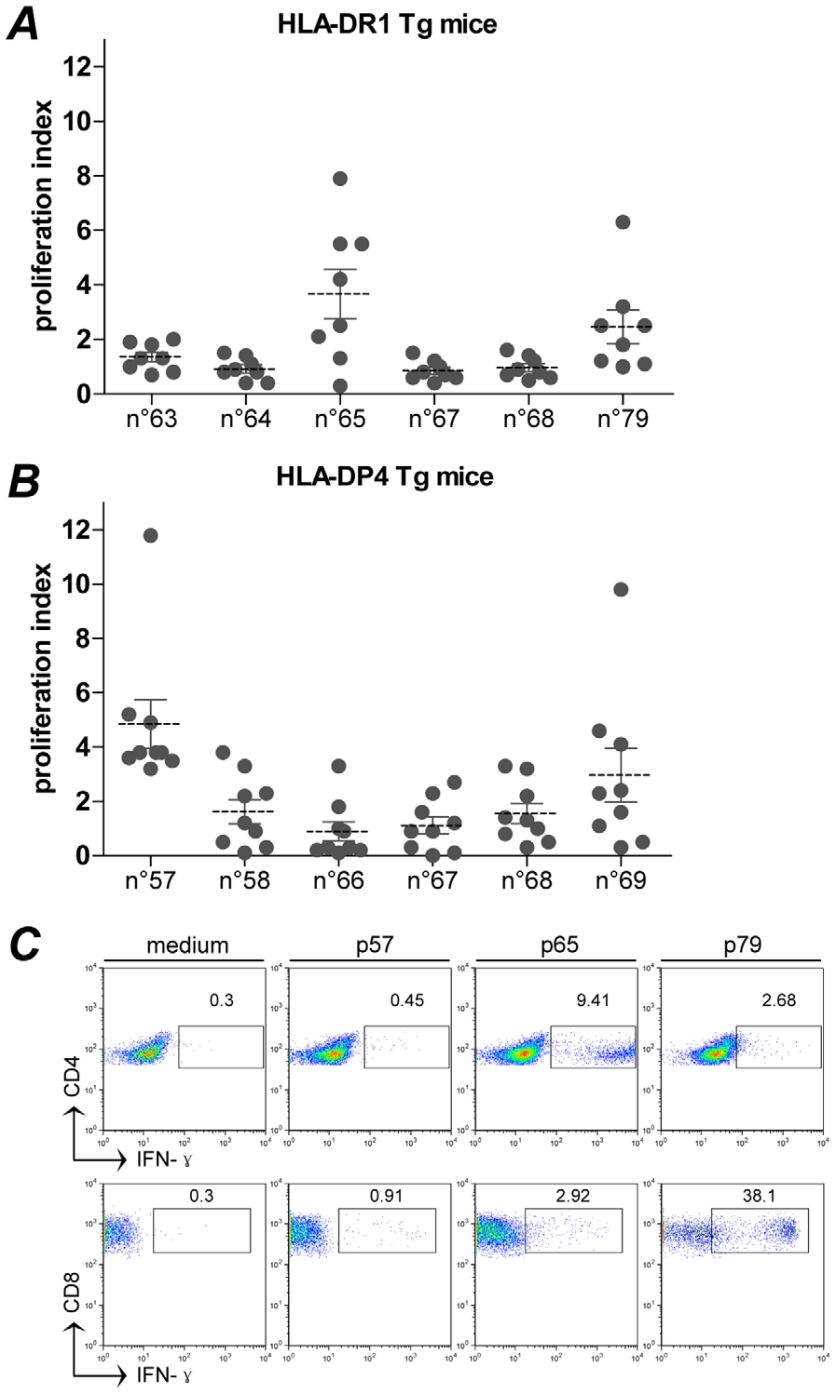
Evaluation of the T cell response in transgenic mouse models following gWiz-F immunization. **A**, **B**, splenocytes proliferation assay. Splenocytes from immunized HLA-DR1 (n = 8) (**A**) or -DP4 (n = 9) (**B**) transgenic mice were stimulated individually with peptides spanning the F protein as described in Materials and Methods. Only six peptides represent in each plot. Responses are expressed as a proliferation index. Individual data are shown by the circular plots. The dotted lines correspond to the mean values of the stimulation index for each group (>2 considered positive). **C**, intracellular staining of IFN-γ. Splenocytes from a representative HLA-DR1 mouse receiving gWiz-F immunization were re-stimulated by p57, p65 and p79 respectively. CD4^+^ or CD8^+^ lymphocytes were specially gated for the IFN-γ detection.

We first investigated whether the MHC class II binding determinants of HCV F protein could specifically stimulate CD4^+^ T cell response by DNA vaccination in humanized mouse models. The transgenic mice expressing the human HLA-DR1 or HLA-DP4 molecules [18] were intramuscularly immunized twice with gWiz-F. 7 days after the second injection, splenocytes from immunized mice were isolated and subjected to *in vitro* proliferation assays co-cultured with F protein-derived 15-mer peptides individually (**Table 1**). As shown in **Fig. 2**, after the stimulation with some F protein derived peptides, the splenocytes experienced a significant *in vitro* expansion (p65 for HLA-DR1 mice, and p57, p69 for HLA-DP4 mice). p65 was frequently recognized (7/8) by HLA-DR1 mice, while 5/9 and all the tested HLA-DP4 mice elicited p69- or p57-specific immune response respectively. p58, the overlapping peptide adjacent to p57, could also be reactive to the PBMCs from several HLA-DP4 mice although the proliferative responses were less significant compared to p57 stimulation (**Fig.2B**).

**Table 1:** The sequences of synthesized HCV F protein peptides.*

Number	Sequence	Number	Sequence
57	GPGWVCARLGRLPSG	67	WGGQDGSCHPEAPGL
58	CARLGRLPSGRNLVE	68	GSCHPEAPGLVGAPQ
59	RLPSGRNLVEGDNLS	69	EAPGLVGAPQTPGVG
60	RNLVEGDNLSPRLAV	70	VGAPQTPGVGRVIWV
61	GDNLSPRLAVPRAGP	71	TPGVGRVIWVRSSIP
62	PRLAVPRAGPGRSPG	72	RVIWVRSSIPSHAAS
63	PRAGPGRSPGTLGPS	73	RSSIPSHAASPISWG
64	GRSPGTLGPSMAMRA	74	SHAASPISWGTFRLS
65	TLGPSMAMRAWGGQD	75	PISWGTFRLSAAPLG
66	MAMRAWGGQDGSCHP		

*Totally 37 overlapping peptides covering the chimeric HCV F protein were synthesized. Only the peptides from the central frameshift F domain (aa43-144) are shown.

The peptide p79 (LAHGVRVLEDGVNYA), located at the common C-terminal region of HCV F and the parental core protein, was shown as well in our study as a potential epitope to stimulate a significant proliferation response in HLA-DR1 mice (**Fig. 2A**). By intracellular and surface staining, however, the p79 activated cells were mostly identified as CD8^+^ lymphocytes, whereas p65 induced IFN-γ-producing cells were stained as the CD4^+^ population. As shown in **Fig. 2C**, the IFN-γ-producing cells even represented as 9.41% of CD4^+^ lymphocytes following p65 re-stimulation. Although p57 and p69 elicited significant proliferative responses of splenocytes from HLA-DP4 mice following DNA immunization, the peptides failed to stimulate significant IFN-γ production in the *ex vivo* intracellular staining assay (data not shown) which might be due to a low frequency of the specific T cells presented in the mice.

Taken together, our study in HLA-DR1 and -DP4 transgenic mice revealed that HCV F protein could elicit HLA-class II restricted CD4^+^ T cell response *in vivo*. Several stimulating peptides within HCV F protein were identified as HLA-DR or HLA-DP presenting epitopes.

### F protein elicited specific CD4^+^ T cell response in chronic infected patients

In order to identify HCV F protein-specific HTL responses during HCV natural infection, we firstly recruited a cohort of 47 patients with HCV chronic infection and a control group of 22 healthy subjects in our study. HLA-DR and -DP genotyping were assayed by sequence-specific-primer PCR (Polymerase chain reaction) using extracted genomic DNA as templates. 5 patients and 3 control subjects were finally identified as HLA-DP*0401, whereas only one patient and one healthy control expressed the MHC class II allele of HLA-DRB1*01 (**Table 2**).

**Table 2:** Analysis of the sero-prevalence of anti-HCV antibodies by ELISA in HCV chronically infected patients.

HLA-genotype	Number	Anti- a		
		F peptide b	Core protein	c22-3/c200/NS5
Total^c^	62	22/62 (35%)	57/62 (92%)	62/62 (100%)
HLA-DR*01	1	1/1	1/1	1/1
HLA-DP*0401	5	2/5	4/5	5/5
Not tested	15	5/15	13/15	15/15

a , values of the Elisa assay are presented as the number of positive patients versus patients tested, with calculated percentages in the parentheses;

b , a synthetic 99 aa F peptide spanning the central frameshift F domain;

c , the HLA class II haplotypes including HLA-DRB1*01, 03, 04, 07, 08, 09, 10, 11, 12, 13, 14, 15, 16, and HLA-DP*0401 negative or positive subtypes.

**Figure 3:**
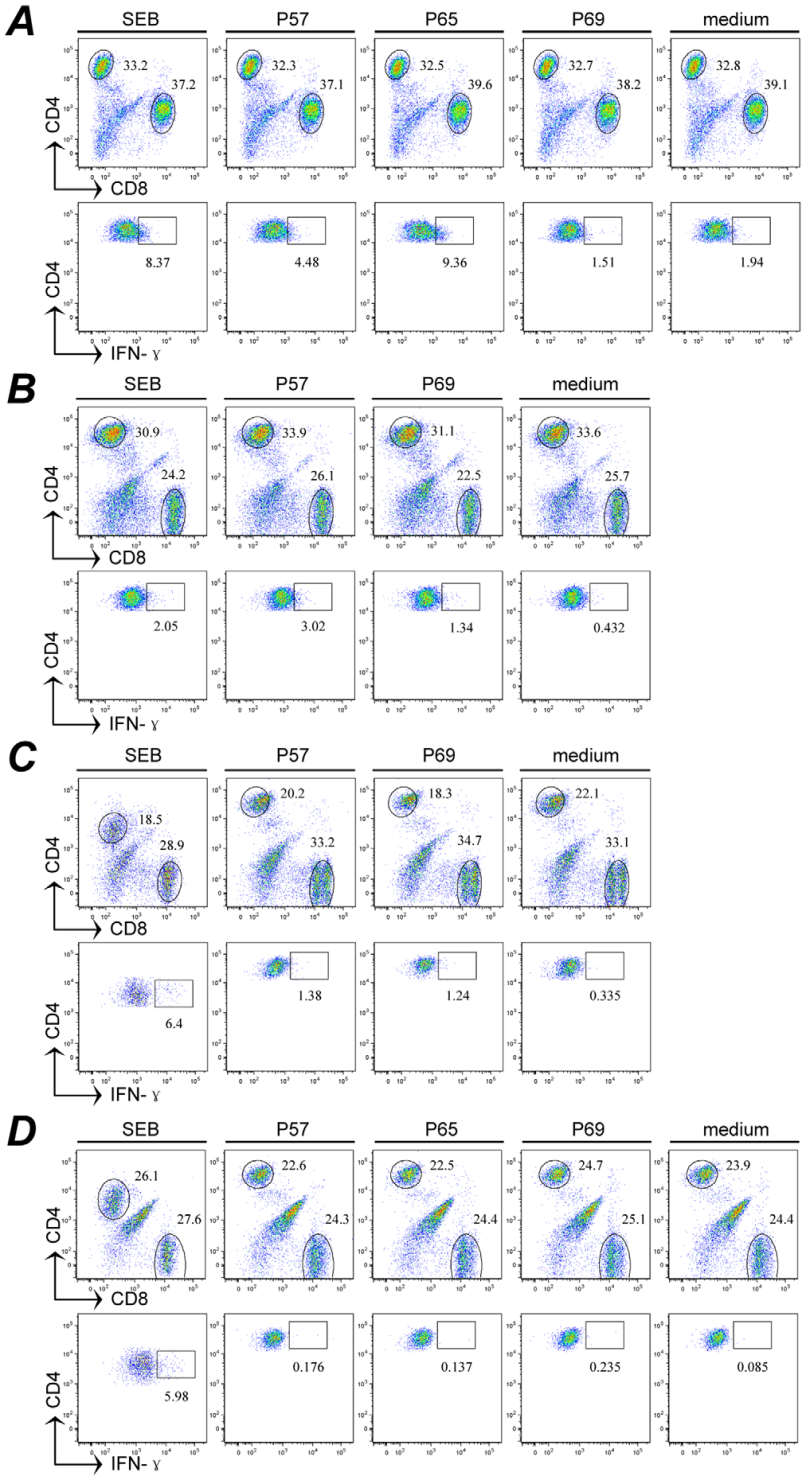
F protein elicited specific HTL responses in patients with chronic hepatitis C. Isolated PBMCs from HLA-DR1 and -DP4 subjects were co-cultured with a peptide pool containing p57, p65, and p69. After 10 days *in vitro* expansion, cells were harvested to test IFN-γ production re-stimulated by cognate peptides. With the surface and intracellular staining, CD4^+^ T cells were analyzed by flow cytometry. **A**, the PBMC from a HLA-DR1 patient; **B**, **C**, PBMCs from two HLA-DP4 patients. **D**, a representative healthy control of HLA-DP4 genotype. The percentage of CD4^+^ IFN-γ cells is indicated in the upper right quadrant of each plot. SEB, staphylococcal enterotoxin B. Single peptides that elicited distinct responses of more than two times background (medium) IFN-γ production are considered positive.

Freshly isolated PBMCs from HLA-DR1 and -DP4 subjects were further incubated in the presence of a mixed peptide pool (p57, p65, and p69). After 10 days of *in vitro* expansion, cells were harvested to test IFN-γ production following the re-stimulation of the cognate peptides (p65 for HLA-DR1, p57 and p69 for HLA-DP4 subjects). In the patient of HLA-DR1 genotype, HCV-F specific CD4^+^ T cell response against p65 (TLGPSMAMRAWGGQD) was exhibited by intracellular cytokine staining as shown in **Fig. 3A**. The percentage of IFN-γ-producing cells represented up to 9.36% of CD4^+^ T cells in the presence of p65 stimulation, compared with 8.37% by SEB and 1% in the absence of stimulation respectively. In the 5 patients of HLA-DP4 genotype, p57 stimulated CD4^+^ T cell activation only in the PBMCs of two subjects (3.02% and 1.38% of CD4^+^ T cells respectively, **Fig. 3B, C**). p69 induced-specific production of IFN-γ was also observed in the two subjects, but to a less extent (1.34% and 1.24% respectively). In the healthy control with HLA-DR1 or -DP4 genotype, the three peptides did not stimulate obvious IFN-γ production by the PBMCs after *in vitro* expansion.

We further analyzed the 3 HCV patients who exhibited the F protein specific T cell response. According to the clinical investigation, the three patients displayed lower viral copies (3.62×10^4^, 5.38×10^5^, and 2.81×10^4^ copies/ml) and mild alanine transaminase levels (96, 102, and 132 IU/ml) in the sera before antiviral treatments, compared with the mean viral load of 3.32×10^6^ copies/ml and transaminase level of 115 IU/ml respectively of the total recruited patients. We thus proposed that the F protein specific HTL response, even only at a detectable level, might reflect a favorable state for the antiviral immunity of the hosts. Nevertheless, no obvious preference for the antiviral treatments was observed in the three patients. Actually, most recruited patients had a remarkably improved viral control following antiviral treatments or even a spontaneous resolution.

In addition to our first try in 47 HCV patients, a new cohort of 15 more patients has been recruited for testing the F protein-specific HTL response. Considering the low incidence of HLA-DR1 and -DP4 haplotypes in HCV patients, three pools of mixed peptides spanning the central F frameshift domain (pool A, p57- p62; pool B, p63–p68; and pool C, p69–p75) were used directly for the *in vitro* expansion of freshly isolated PBMCs from these patients, regardless of the HLA class II subtypes. After re-stimulation with the mixed peptides, although some activation could be observed in several PBMC samples, they were not strictly significant according to the assay of IFN-γ intracellular staining (data not shown). Moreover, the humoral immune response against HCV was examined in the recruited patients (**Table 2**). Interestingly, the three subjects who exhibited F protein specific CD4^+^ T cell response were all anti-F antibody positive by the ELISA assays. It is also noteworthy that an overall 22 of tested patients (35%) displayed anti-F antibodies in the sera although most of them did not show the HLA-DR1 or -DP4 restricted T cell responses. Taken together, the current study provided the evidence that HCV F protein specific CD4^+^ T cell response could be induced in HCV chronically infected patients, although the potency and width of the HTL response were relatively low. The hyporesponsiveness to HCV F protein might reflect a progressive dysfunction of specific T cell responses during the chronic viral infection.

## Discussion

Bain and colleagues previously identified a specific CD8^+^ T cell response against the HCV F protein which was expressed from an alternate reading frame of the viral genome during HCV natural infection [19]. Recently, we and others have further demonstrated that the specific antibodies against the F protein presented in HCV chronically infected patients [16], [17]. However, the specific CD4^+^ T cell responses against the F protein during HCV infection and the pathological implications remain unclear. CD4^+^ T cells can differentiate into Th1, Th2, Th17, and regulatory T cell subsets, whose different immunological functions are associated with the production of particular cytokines that play important roles during HCV infection. Interestingly, Drouin C *et al* [20] reported more recently that cell-mediated immune responses directed against HCV F protein were undetectable during the acute viral infection. In the current study, comparatively, we screened the MHC class II-presenting epitopes of the F protein through HLA-transgenic mouse models and eventually validated the specific CD4^+^ T cell responses in HCV chronically infected patients.

The antigen-processing machinery is highly conserved in mouse and human cells [21]. HLA-transgenic mice with knockouts of murine MHC molecules have proven to be an excellent preclinical model for the characterization of epitopes relevant to T cell recognition in humans. In this study, we constructed the gWiz-F as a fusion gene vector which could produce the F protein in eukaryotic cells as indicated by western blot assay. gWiz-F was further applied by DNA vaccination which made it possible to assess the immune activity of F protein in HLA-transgenic mice *in vivo*. Historically, the proliferative response of T cells to mitogenic challenge has been a good standard for assessing T cell function in both clinical and investigative immunology. In our study, three potential HLA-DR1 or -DP4 presenting epitopes were eventually defined by the splenocyte proliferation assay with humanized mouse models.

We next tried to identify HLA-matched subjects in order to characterize the specific CD4^+^ T cell response against the F protein during HCV natural infection. The HLA class II–restricted T cell response to hepatitis C virus (HCV) antigens is believed to influence the final outcome of HCV infection [22]. HLA-DRB1*01 allele appears to be related to a better HCV specific T cell response according to the previous report by Barrett S *et al* [23], and is thus with a rare incidence in the patients with HCV chronic infection. In our study, we successfully recruited some HCV patients with HLA-DP*0401 or HLA-DRB1*01 alleles. With the PBMCs from those patients, we eventually validated the epitopes and the CD4^+^ T cell response defined by the experiments with transgenic mice.

Tsai *et al* [24] previously demonstrated that viral clearance was more likely to occur when PBMCs of acute hepatitis C patients displayed a Th1 profile (IFN-γ and IL-2) upon stimulation with HCV antigens than in patients developing a Th2 phenotype (IL-4 and IL-10). Even in the chronic infection, a strong Th1 response seems to be associated with a less inflammatory course of the disease [25]. Therefore, a Th1 profile may be considered as generating more protective immune responses in HCV infection and in contrast a failure to induce this phenotype may contribute to the evolution to chronicity. In our study, the overlapping 15-mer peptide pools spanning the HCV F sequence were used for the PBMCs expansion *in vitro* to characterize the IFN-γ production by peptide re-stimulation individually. The three HCV F peptides activated INF-γ producing response in some patients with well-matched HLA class II subtype respectively. It is noteworthy that, although eventually five HCV patients were identified with the proper HLA-DP4 haplotype, they did not commonly exhibit the CD4^+^ T cell response against the defined peptides. This might reflect the difference between the immune competent naïve state in mouse models and the specific immune hyporesponsiveness during the natural HCV persistent infection.

Analysis of HCV-specific CD4^+^ T cell responses in chronic HCV infection using Elispot or intracellular staining showed responses at low frequency in blood and only targeted a limited number of epitopes [18], [26], [27], [28]. Recent data also suggest that, in the presence of viraemia, HCV-specific CD4^+^ T cell populations do exist but lack proliferative capacity [29]. These may explain the hyporesponsiveness of PBMCs upon F peptide stimulation observed in patients with chronic hepatitis C. Additionally, the geographical regions, races of the patients, HCV genotypes, and sample sizes could have impacts on the investigation. The very limited proportion of Chinese HCV patients with HLA-DP*0401 and HLA-DRB1*01 genotypes is a significant restriction factor of the current study.

In summary, HCV F protein, the alternative reading product of the viral genome, is expressed during the chronic viral infection that stimulates specific immune responses *in vivo*. Our study directly evidenced the specific CD4^+^ T cell responses against HCV F protein in transgenic mice following DNA immunization, and also in patients chronically infected with HCV. Although the F protein induced potent CD4^+^ T cell responses with defined epitopes in HLA-transgenic mice, these specific T cell responses were less obvious in HCV chronically infected patients. The hyporesponsiveness to HCV F protein might reflect a progressive dysfunction of specific T cell responses during the chronic viral infection. However, the study of the HCV F protein is still at an early stage. Clearly, more investigations of the relationship between anti-F immune response and the pathogenesis of Hepatitis C virus infection are warranted.

## Materials and Methods

### Ethics Statement

The current research involving human participants has been approved by the institutional review board of Shanghai Ruijin Hospital with written consents. All experiments involving mice were performed according to approved protocols and guidelines of the animal facility of Hôpital Paul Brousse (Villejuif, France) with the agreement number 94-076-32, and the permit number 94–241 from Direction des Services Vétérinaires.

### Animal studies

The HLA-A2/-DR1, and HLA-A2/-DP4 transgenic mice (H-2 class II knockout, HLA-A*0201 transgenic, and HLA-DRB1*0101 or -DP*0401 transgenic) [18] were bred in the animal facility of Hôpital Paul Brousse. Groups of 6–10 weeks old female mice received two vaccinations, 2-week apart, of gWiz-core or gWiz-F. Intramuscular DNA immunizations were performed under anesthesia by injecting 100 µg (1 mg/ml) of plasmid DNA into regenerating (i.e. cardiotoxin-treated) tibialis anteriormuscles [30].

### Peptides

A total of 37 peptides covering the entire 191 aa of HCV F protein were synthesized by Gelson Chemical (Shanghai, China). These peptides were 15 aa in length, overlapping adjacent peptides by 10 aa. The sequence of individual peptide from the central frameshift F domain (aa43-144) of the HCV F protein is listed in **Table 1**. Stock solutions of all synthetic peptides were produced at 1 mg/ml in either water or 20% DMSO, according to the supplier's instruction. The peptides were used for the *in vitro* co-culture and the re-stimulation of splenocytes. All the peptides spanning the chimeric HCV F protein (**Table 1**) were tested by the splenocytes proliferation assay. Peptides with the potential avidity were further verified by a second round of screening.

### Plasmid construction

HCV core gene was PCR amplified from the viral cDNA after reverse transcription using the serum sample from genotype 1b HCV infected patients. The HCV F protein-coding sequence was achieved by the +1/−1 frame-shifting between codon 43 and codon 144 of the core gene by overlapping PCR. The resulting HCV F cDNA was subcloned into the gWiz expression vector (Genlantis, San Diego, CA) to have gWiz-F. The plasmid of gWiz-core encoded HCV core gene (genotype 1b) driven by the cytomegalovirus (CMV) early gene promoter.

### *In vitro* transfection assay

Human hepatoma Huh 7 cells [31] were maintained in DMEM containing 10% fetal calf serum. Cells were harvested 48 h after Lipofectamine 2000 transfection (Invitrogen). Cell lysates were analyzed by Western-blot according to standard procedures, with anti-core and anti-HCV F monoclonal antibodies (CNRS, France). Signals were visualized using the enhanced chemiluminescence method with a horseradish peroxidase-labeled rabbit anti-mouse immunoglobulin (Dako, Carpenteria, CA).

### Proliferation assay of mouse splenocytes

7 to 10 days after the second DNA injection, mouse spleen was smashed with a syringe plunger in a 70 µm cell strainer (100 µm Nylon, BD). Red blood cells were removed by Ficoll gradient centrifugation. Isolated splenocytes (106 per well) were incubated with 10 µg/ml peptide for 3 days in supplemented HL1 serum-free medium and pulsed for the final 16 h with 1 µCi (3H)-thymidine per well [30]. All the peptides spanning the chimeric HCV F protein (**Table 1**) were measured by the splenocytes proliferation assay. Peptides with the potential avidity were further verified by a second round of screening.

### Patient cohort

Totally sixty two subjects of chronic HCV infection with written informed consent were enrolled in this study from the Department of Infectious Diseases of Shanghai Ruijin Hospital. No subject had been treated with antiviral therapy before this study. The diagnosis of chronic hepatitis C was based on internationally accepted criteria [32]. All subjects were anti-HCV positive as measured by Third-Generation Enzyme Immunoassay. The Mean HCV load (copies/ml) was around 3.32×10^6^ (range from 2.3×10^4^ to 2.2×10^8^) quantified in serum samples by real-time PCR after reverse transcription of the 5′noncoding region of the HCV genome (PJ Co. Ltd., China). HCV genotypes were determined using Realchip Kit (Realchip Biotechnology, China) by which 66% of the samples were of HCV-1b subtype. The average alanine transaminase (ALTs) level was 115 IU/ml (range from 16 to 851 IU/ml).

### Genotyping of HLA class II alleles

The genomic DNA was extracted from patients' PBMCs using the Puregene DNA isolation kit for blood (Qiagen). HLA-DR and HLA-DP genotyping were carried out by primer specific PCR using Olerup SSP Genovision kit (Sweden).

### *In Vitro* Expansion of the PBMCs

PBMCs were isolated from fresh heparinized blood by Ficoll density gradient centrifugation and seeded at 5×106 cells/well in 24-well plates with RPMI 1640 complete medium containing 2 mM L-glutamine, 1 mM sodium pyruvate, nonessential amino acids, 100 U/ml penicillin, 100 µg/ml streptomycin, and 10% FCS (Gibico). Cells were stimulated by incubation with HCV F peptide pools (1 µg/ml of each peptide). Half the medium was replaced every 3 days with complete medium supplemented with recombinant IL-2 (50 IU/ml) (Roche). After 10 days of culture, HCV F-specific IFN-γ-producing cells were quantified by intracellular staining and flow cytometry analysis.

### Intracellular staining assay

Freshly isolated PBMCs were incubated overnight either with 500 ng/ml staphylococcal enterotoxin B (SEB, Sigma) as a positive control, or with individual HCV F-derived peptides (1 µg/ml) in the presence of 2 µg/ml brefeldin A (BFA, Sigma). Cells were suspended in PBS containing 1% BSA and labeled with anti-CD4-APC (clone HIT3a) and anti-CD8-FITC (clone RPA-T4) for 20 minutes at 4°C. After fixation and permeabilization, cells were further stained with anti-human IFN-γ-PE (clone 4S.B3) for 30 minutes at 4°C. At least 50,000 lymphocyte-gated events were acquired on a FACSCalibur flow cytometer (BD Biosciences) and analyzed using Flowjo software (version 8.7.3, Tree Star). Conjugated antibodies were purchased from BD Biosciences. Background staining was assessed with an isotype-matched control monoclonal antibody. Single peptides that elicited distinct responses of more than two times background IFN-γ production were considered positive [33].

### Enzyme-linked immunosorbent assay (ELISA)

The ELSIA assay testing the humoral immune response in sera of HCV patients was described elsewhere [17]. A synthetic 99 aa F peptide encoding the central frameshift F domain was synthesized by Gelson Chemical. The recombinant 6×His tagged HCV core protein were prokaryotically expressed and purified according to our previous work. In addition, antibody to hepatitis C virus in these patients were detected as controls by using hepatitis C Virus encoded Antigen (Recombinant c22-3, c200 and NS5) with ORTHO HCV Version 3.0 ELISA Test System (Ortho-Clinical Diagnostics) according to the manufacturer's recommendations.
